# The Virtuous Servant Owner—A Paradigm Whose Time has Come (Again)

**DOI:** 10.3389/frobt.2021.715849

**Published:** 2021-09-22

**Authors:** Mois Navon

**Affiliations:** Department of Jewish Philosophy, Bar Ilan University, Ramat Gan, Israel

**Keywords:** social robots, artificial intelligence, ethics, jewish thought, virtue, slave

## Abstract

Social Robots are coming. They are being designed to enter our lives and help in everything from childrearing to elderly care, from household chores to personal therapy, and the list goes on. There is great promise that these machines will further the progress that their predecessors achieved, enhancing our lives and alleviating us of the many tasks with which we would rather not be occupied. But there is a dilemma. On the one hand, these machines are just that, machines. Accordingly, some thinkers propose that we maintain this perspective and relate to Social Robots as “tools”. Yet, in treating them as such, it is argued, we deny our own natural empathy, ultimately inculcating vicious as opposed to virtuous dispositions. Many thinkers thus apply Kant’s approach to animals—“he who is cruel to animals becomes hard also in his dealings with men”—contending that we must not maltreat robots lest we maltreat humans. On the other hand, because we innately anthropomorphize entities that behave with autonomy and mobility (let alone entities that exhibit beliefs, desires and intentions), we become emotionally entangled with them. Some thinkers actually encourage such relationships. But there are problems here also. For starters, many maintain that it is imprudent to have “empty,” unidirectional relationships for we will then fail to appreciate authentic reciprocal relationships. Furthermore, such relationships can lead to our being manipulated, to our shunning of real human interactions as “messy,” to our incorrectly allocating resources away from humans, and more. In this article, I review the various positions on this issue and propose an approach that I believe sits in the middle ground between the one extreme of treating Social Robots as mere machines versus the other extreme of accepting Social Robots as having human-like status. I call the approach “The Virtuous Servant Owner” and base it on the virtue ethics of the medieval Jewish philosopher Maimonides.

## Introduction

“Man is by nature a social animal” (*Politics*, 1253a). So noted Aristotle almost 3,000 years ago. Interestingly, while Aristotle did actually conceptualize automatons that might replace the slave labor of his day (ibid., 1253b), he did not envision that humans might interact socially with these automatons. This is because, in addition to living at a time when human slaves were considered animated tools, he never imagined the sophisticated automatons of the twenty-first century—i.e., social robots, which today come in a vast and growing array of configurations (Reeves et al., 2020), many designed to be social companions.^1^ Indeed, the social robots of today are not merely functional automatons, they are emotionally engaging humanoids. And even those not designed to be so, nevertheless manage to trigger our empathy, drawing us to relate to them *as if* they too were, by nature, a “social animal.”

It is this “as if” (Gerdes, 2016: 276) condition that brings us to one of the most consternating conundrums in the field of robo-ethics today, what Mark Coeckelbergh calls, “the gap problem” (Coeckelbergh, 2013; Coeckelbergh, 2020c). When we interact with a Social Robot (SR), a “gap” exists between what our reason tells us about the SR (i.e., it is a machine) versus what our experience tells us about the SR (i.e., it is more than a machine). It is this gap that gives rise to the ethical question that is the subject of this essay: How are we to relate *morally* to social robots—like a machine or more than a machine?

Before attempting to address this question, it is important to define specifically the type of SR that is the focus of this investigation. Social robots are currently powered by artificial intelligence, which enables them to “learn” from their experiences, modify their behavior accordingly, and give the appearance of autonomy—the appearance of beliefs, desires and intentions. These features are the hallmarks of consciousness and what make us, in large part, who we are. But today, the artificial intelligence powering our social robots is entirely artificial—entirely based on mathematics (see, e.g., Domingos, 2018; Boucher, 2019; Brand, 2020: 207; Coeckelbergh, 2020a: 83–94)^2^—the robot only behaves *as if* it has consciousness.

There are hopes, even designs, to make social robots with true human-like second-order consciousness—i.e., to make a sentient, self-aware being that has the capability to think about its own thoughts. However, while this may be the ultimate goal of the AI project, what Ray Kurzweil calls “the singularity,” its achievement remains a long way off (see, e.g., Torrance, 2007: 500; Coeckelbergh, 2010a: 210; Wallach and Allen, 2010: 8; Tallis, 2012: 194; Veruggio and Abney, 2012: 349; Prescott, 2017: 5; Sparrow, 2017: 467; Bertolini and Arian, 2020: 45; Birhane and van Dijk, 2020: 210; Hauskeller, 2020: 2). And even the less ambitious HLMI [High-Level Machine Intelligence] is a long way off, see, e.g., Grace et al. (2018), Boucher (2019: 10), and Shalev-Shwartz et al. (2020: 2). Some, however are optimistic: Dyson (2012), Moravec (1988), Kurzweil 1999 cited in Sparrow and Sparrow (2006), Long and Kelley 2010, O’Regan 2012, and Gorbenko et al. 2012 cited in Neely (2013). Accordingly, this paper does not seek to discuss social robots with human-like consciousness, nor even with simple animal sentience,^3^ but rather social robots that are driven by current day artificial intelligence—i.e., robots that are essentially autonomous mobile computers with humanlike physical characteristics,^4^ what I call: mindless humanoids.

## The Dilemma

So, again, the question is: How are we to relate *morally* to social robots?

In general, when we encounter a new entity—be it mineral, vegetable, animal, or human—we seek to categorize it according to its various ontological properties (see, e.g., Coeckelbergh, 2013: 63; Johnson and Verdicchio, 2018: 292). We do this so that we know how to interact with it, and more profoundly, how to interact with it morally. For example, if it is a rock, we know we can kick it into an open field without qualms about harming the rock; if it is a neighborhood cat, we know that we shouldn’t kick it or otherwise indiscriminately cause it pain; if it is our human co-worker, we realize that greater moral consideration is due him than a cat. In short, we ask what the entity “is” in order to determine how we “ought” to treat it.^5^ This approach is variously known as the ontological approach, the properties approach (Tavani, 2018), the mind-morality approach (Gerdes, 2016), the organic approach (Torrance, 2007; Tollon, 2020), the realist approach (Torrance, 2013) or simply, the standard approach (Coeckelbergh, 2013).

The ontological approach, however, encounters difficulties with social robots as they fall into a strange middle ground between man and machine, presenting the previously mentioned gap problem, alternatively referred to as a “category boundary problem” (Coeckelbergh, 2014: 63). On the one hand, the SR is a mindless automaton, programmed^6^ to carry out various social tasks—i.e., a machine. On the other hand, the SR, designed with human-like physical characteristics and programmed to carry out its tasks with human-like behavior, appears to us as, well, human-like. Furthermore, even if we are aware of the fact that it is not human, that it does not have a mind, a consciousness, we are nevertheless deceived (see, e.g., Turkle, 2011a: 63, 90; Grodzinsky et al., 2014: 92, 98; Richardson, 2015: 124; Gunkel, 2018: 115; Leong and Selinger, 2019: 307).

The deception is of course self-deception, a result of our own human “programming,” if you will. We are “wired” to respond to animacy, to self-propelled entities that “make eye contact, track our motion, and gesture in a show of friendship” (Turkle, 2011a: 8; see also, e.g., Arico et al., 2011; Gray and Schein, 2012: 408; Scheutz, 2014b: 213; Darling et al., 2015: 770; Schwitzgebel and Garza, 2015: 112; Darling, 2016: 217; Ghiglino and Wykowska, 2020: 53). These behaviors push, what Sherry Turkle calls, “our Darwinian buttons,” inducing us to ascribe human attributes to such robots until we “imagine that the robot is an ‘other,’ that there is, colloquially speaking, ‘somebody home’” (Turkle, 2011a: 8; see also, e.g., Foerst, 2009; Arico et al., 2011; Turkle, 2011b: 63; Scheutz, 2014b: 215; Richardson, 2015: 72; Bertolini, 2018: 649; Fossa, 2018: 124). Sven Nyholm calls this “mind reading”—we read into the behaviors of others their apparent mental state, their mind (Nyholm, 2020; see also, e.g., Richardson, 2015: 74; Darling, 2016: 216; de Graaf and Malle, 2019; Ghiglino and Wykowska, 2020: 51). Others (e.g., Duffy, 2003: 180; Huebner, 2009; Veruggio and Abney, 2012: 355; Ghiglino and Wykowska, 2020: 67; Tollon, 2020: 7) say we adopt, what Daniel Dennett terms, the “intentional stance,” whereby we treat an entity “*as if* it were a rational agent who governed its ‘choice’ of ‘action’ by a ‘consideration’ of its ‘beliefs’ and ‘desires’” (Dennett, 1996).

This phenomenon of seeing social robots as humanlike is known as anthropomorphism, but it doesn’t end with simply ascribing human beliefs, desires and intentions to the robot—we take it to the next step and become engaged, emotionally, with the social robot (see, e.g., Coeckelbergh, 2009; Choi, 2013; Grodzinsky et al., 2014: 92; Darling, 2016: 214; Richards and Smart, 2016: 18; Darling, 2017; Johnson and Verdicchio, 2018; Tavani, 2018: 3; Gunkel and Wales, 2021; see also sources cited in previous paragraph). And this engagement isn’t just some kind of fictional role playing, but rather, we feel real empathy toward the social robot (see, e.g., Redstone 2014; Darling et al., 2015; Wales, 2020). Indeed, Tony Prescott notes that “we do not need to believe (or be deceived) that the psychological states, intentional, or phenomenological, that we read into an artefact, such as a robot, are akin to our own in order to experience an authentic and meaningful emotional response” (2017: 144).

Now, while this emotional anthropomorphizing is going on, another socio-psychological element comes into play: dehumanization. Massimiliano Cappuccio et al., describe this troubling phenomenon:

“… the fundamental ethical problem at the core of social robotics is that, while robots are designed to be like humans, they are also developed to be owned by humans and obey them. The disturbing consequence is that, while social robots become progressively more adaptive and autonomous, they will be perceived more and more as slave-like. In fact, owning and using an intelligent and autonomous agent instrumentally (i.e., as an agent capable to act on the basis of its own decisions to fulfill its own goals) is precisely the definition of slavery” (Cappuccio et al., 2019: 25).

Cappuccio et al. call this the Anthropomorphism Dehumanization Paradox (ADP). Jordan Wales (2020) calls it “the dilemma of empathy and ownership,” explaining that if we allow ourselves to engage emotionally with robots, we will nevertheless use them for what we acquired them to do and, accordingly, end up treating them as slaves (similarly, Walker, 2006). This might not seem so terrible since the machine “feels” no indignity or ignominy, no disgrace or denigration—indeed, the machine “feels” nothing.^7^ The problem, however, is not for the machine but for man, as Kant famously noted:

So if a man has his dog shot, because it can no longer earn a living for him, he is by no means in breach of any duty to the dog, since the latter is incapable of judgement,^8^ but he thereby damages the kindly and humane qualities in himself, which he ought to exercise in virtue of his duties to mankind. Lest he extinguish such qualities, he must already practise a similar kindliness towards animals; for a person who already displays such cruelty to animals is also no less hardened towards men. We can already know the human heart, even in regard to animals (Kant, 1996, 212).^9^

Similarly, it is feared that our instrumental treatment of human-like robots—treating them as slaves—will then influence our treatment of humans (e.g., Levy, 2009; Anderson, 2011: 294; Darling, 2016: 227–8; Cappuccio et al., 2019: 14; Chomanski, 2019: 1008; Gunkel and Wales, 2021: 4, 9; Coeckelbergh, 2021: 7; in opposition see, e.g., Johnson and Verdicchio, 2018; Bryson, 2020a: 22). We will likely not treat people as slaves, but we will certainly be in danger of treating people as objects rather than subjects. Our relationships with SRs, to put it Buberian terms, could be seen as habituating an I-It relationship as opposed to cultivating an I-Thou relationship (Buber, 1970). The SR would thus invert Buber’s call to relate to the other as subject not object, hardening us, to echo Kant, to view the other as object not subject (Hawley, 2019, 12). And this, ultimately, reflects upon the individual as vicious as opposed to virtuous.^10^ For Buber, the individual—the “I”—is not merely influenced by his relationship with the other, he is *defined* by it. “There is no I as such but only the I of the basic word I-Thou and the I of the basic word I-It. When a man says I, he means one or the other” (Buber, 1970: 54). Consequently, some, like Michael Burdett (2020), have suggested that it would be appropriate for us to relate to a robot as a “Thou.” Others, like Elizabeth Green (2018) argue that a robot can never be a Thou, while still others, like Sherry Turkle (2011a: 85), explain that the “Thou” relationship simply emerges.

## Resolutions

This brings us into the thick of possible “resolutions” to the dilemma. I keep the term “resolution” in quotes because this dilemma, like all worthy of the name, only reach resolution with the sacrifice of ideals. This point will be made all too clear in the following review of proposed resolutions.

Returning to Cappuccio et al. (2019: 26), who describe the dilemma as a paradox, we encounter two practical approaches to dissolve the paradox: either reduce—by design—the elements that promote anthropomorphizing, thus keeping the machine very much a machine,^11^ or conversely, increase those elements that engender empathy to encourage human to human-like interaction.^12^ Both approaches, they note, are not really solutions. Reducing the anthropomorphic elements of SRs undermines their very purpose as companions that are to “establish trust and cooperation, [be it] with a child, a patient with disabilities, or an elderly person” (Cappuccio et al., 2019: 26). On the other hand, increasing such elements that engender human-like empathic relationships, opens a Pandora’s box of ethical issues based on the misperception of the true nature of the machines, including but not limited to: developing intimate relationships with robots (Turkle, 2011a: 295; Richardson, 2015: 12; Gerdes, 2016: 277; Bertolini, 2018: 653), shunning human relationships as “messy” (Turkle, 2011a: 7; similarly, Whitby, 2008: 331; Bryson, 2010: 7; Toivakainen, 2015: 10), prioritizing humanoids over humans, thus misspending or misallocating resources (Torrance, 2007: 498; Bryson, 2010: 3; Neely, 2013; Schwitzgebel and Garza, 2015: 114), sacrificing human life (Torrance, 2007: 508; Smids, 2020: 2850), seeing oneself as a machine and thus shirking moral responsibility (Metzler, 2007: 20), and generally maintaining a warped view of reality (Sparrow and Sparrow, 2006: 155; Gerdes, 2016: 276).

The two solutions that Cappuccio et al. float can be seen as an attempt to sway a resolution to the gap problem. That is, either we emphasize what our reason tells us about the SR (i.e., it is a machine) or we emphasize what our experience tells us about the SR (i.e., it is more than a machine). Interestingly, this dichotomy reflects the split of the philosophical community in to two distinct camps.^13^ On the one side, there is the “instrumental” camp, populated by those who believe that machines are machines and, regardless of their appearance and behavior, we should relate to robots like we would to a toaster or a vacuum cleaner (see, e.g., Gunkel, 2018: Ch. 2 “!S1 !S2”). On the other side, there is the “appearances” camp, populated by those who maintain that it is precisely through appearance and behavior that we engage with others and must similarly relate to robots (see, e.g., Gunkel, 2018: Ch. 5 “!S1 S2”).

The instrumental camp could also be referred to as the “insides count” camp, in that they take the position referred to earlier as the “ontological approach.” They derive the moral status of the entity based on its ontology, on “what’s going on inside.” Accordingly, sentience or first-order consciousness is needed for moral patiency and second-order consciousness is needed for moral agency (see, e.g., Anderson, 2013; Smids, 2020). In opposition, the “appearances” camp argues that we have no method to reveal the insides of an entity for we have no “privileged access” to determine if a being is conscious. As a result, we must content ourselves with externals, with the behavior of the entity and its interaction with us. Some here argue that this approach is not simply an accommodation due to epistemological deficiencies but is the philosophically preferred approach based on our lived experience of SRs (see, e.g., Gunkel, 2018; Coeckelbergh, 2010a). Accordingly, we must grant SRs, if not full moral agency then, moral patiency or moral consideration. This approach has been called the relational approach (Coeckelbergh, 2010a; Richardson, 2015) the phenomenological approach (Coeckelbergh 2010b), the hermeneutic approach (Coeckelbergh, 2021), and includes the ethical behaviorist approach (Neely, 2013; Danaher, 2019).

## The Middle Camp

Now, while I have described the dilemma as being approached from two sides, two camps, there is in fact a middle ground, a middle camp, occupied by thinkers that believe insides count but also believe that there are reasons to relate morally to the mindless humanoid as more than a mere machine. That is, though the SR is neither a moral agent nor a moral patient, there are nevertheless ethical demands incumbent upon humans in their interactions with it. Steve Torrance, who I place in this middle camp, describes the moral relationship with a robot as “quasi-moral” (2007: 504, 516). I understand this to mean that the moral demands engendered in the HRR (Human Robot Relationship) do not stem from the inherent moral *status* of the robot but from the relationship, from the moral *implications* of the relationship. This, it should be noted, is in contradistinction to the “relational approach” which sees the mindless humanoid as a “quasi-other.” To be clear, in the “quasi-other” approach it is otherness, alterity, that is imposed on the robot itself which consequently engenders a very real moral demand—e.g., the demand to treat the other like yourself;^14^ whereas in the “quasi-moral” approach, it is morality (e.g., a norm) that is imposed on an otherwise amoral situation.

This quasi-moral approach taken by the middle camp finds its ground in Kant’s indirect duties to the animal kingdom. Kant believed that animals have no moral status and accordingly, he writes, “we have no immediate [i.e., direct] duties to animals; our duties towards them are indirect duties to humanity” (Kant, 1996: 212). Anne Gerdes (2016) explains Kant as teaching that we have not duties *to* animals but rather we have duties *with regard to* animals; similarly, reasons Gerdes (as does Bryson, 2010), we have not duties *to* robots but rather we have duties *with regard to* robots. She brings Kant’s writing on this point in his *Metaphysics of Morals*:

… a propensity to wanton destruction of what is beautiful in inanimate nature … is opposed to a human being’s duty to himself; for it weakens and uproots that feeling in him, which, though not of itself moral, is still a disposition of sensibility that greatly promotes morality or at least prepares the way for it…

With regard to the animate but non-rational part of creation, violent and cruel treatment of animals is far more intimately opposed to a human being’s duty to himself, and he has a duty to refrain from this; for it dulls this shared feelings of their suffering and so weakens and gradually uproots a natural predisposition that is very serviceable to morality in one’s relations with other men. …

Even gratitude for the long service of a horse or dog belongs indirectly to a human being’s duty with regard to these animals; considered as a direct duty, however, it is always only a duty of the human being to himself (6:443).

This passage, as well as the one quoted immediately prior, can be seen as advancing a virtue ethics approach toward non-human entities—as, indeed, Gerdes writes. That is, in our actions toward the inanimate, though no deontological demands bind our behavior, we are nevertheless to refrain from wanton destruction as part our efforts at developing a disposition that promotes moral behavior—i.e., in order to develop our virtuous character (so too, Toivakainen, 2015: 278). With regards to animals, our behavior has an even greater impact on our dispositions. Lara Denis explains that, for Kant, “Any way of treating an animal that could impair our ability to feel love and sympathy for others constitutes a risk to a morally valuable aspect of our rational nature. Kant thinks that cruel or even unloving treatment of animals threatens to impair us in this way” (Denis, 2000: 409). Denis explains that the reason our interactions with animals so affect our dispositions is because we share our animal nature with them and because they engage us emotionally.

Given this, I would argue that, while a SR could be considered an inanimate object, its human-like interaction with us, to the point of our attributing mental states to it, places the SR more closely in the animate category. And though we don’t share our biological animal nature with the robot, we do share behaviors engendered by our animal nature (see, e.g., Turkle, 2011a: Ch. 7). Furthermore, while our emotional engagement with the robot lacks the authentic sentient elements of pain and pleasure characteristic of animal interaction, behaviorally we are just as engaged (see prior sources on emotional engagement as well as, e.g., ibid.; Cappuccio et al., 2019: 15–16). Accordingly, without arguing for the “appearances” approach, I am calling for a virtue approach—i.e., an approach which acknowledges and accounts for how the interaction with a mindless humanoid affects the virtue of the human interlocuter.

The virtue approach to robots is not new and has, in fact, been promoted by numerous thinkers such as: Anne Gerdes (2016), Robert Sparrow (2017, 2020), Shannon Vallor (2018), Massimiliano Cappuccio et al. (2019), and even Mark Coeckelbergh (2020b, 2020c, though he argues against in 2010a). However, while virtue ethics clearly eliminates the “dehumanizing” part of the “anthropomorphizing while dehumanizing paradox,” it would appear to utterly capitulate to the anthropomorphizing part. That is, by relating to the SR in a virtuous manner we avoid the evils inherent in dehumanizing it but remain susceptible to the previously mentioned Pandora’s box of negative consequences associated with anthropomorphizing it. Consequently, Cappuccio et al. (2019: 26) acknowledge that they are thus at a loss to resolve the paradox and content themselves to apply virtue ethics to avoid dehumanizing.

One scholar who does attempt a resolution is Jordan Wales (2020), who employs the thought of Augustine to address the paradox. Augustine, in his *De doctrina Christiana* (1:33:37), teaches that one should ever seek to refer his joy in an other toward God, toward the creator of that individual.^15^ Wales applies this notion to our interactions with SRs, such that, upon feeling natural empathy toward a SR, “we *redirect* that empathy, ‘refer’ it, as Augustine would say, to all the unknown concrete persons whose interactions have unwittingly sculpted the persuasive personality of this instrument” (Wales, 2020: 7). Wales thus solves the anthropomorphism problem, or more precisely, the empathy problem inherent in anthropomorphizing.

To be clear, in anthropomorphizing mindless humanoids, we are in danger of becoming emotionally engaged with entities that do not warrant such engagement and which can thus lead to many social ills (as noted above). By redirecting the empathy in our emotional engagement with the SR toward the real flesh and blood people who served to create it, Wales argues that we avoid attributing humanity to the robot, allowing our emotions to find their proper terminus in true humanity.^16^ As a result, we can interact with the SR in a virtuous way, allowing our natural empathy and anthropomorphizing to occur and yet maintain the realization that the robot is not human, does not have the moral status of a human and does not enter the moral circle of humanity.

Now, while this idea of “referring” or “redirecting” one’s intentions is an accepted notion as a religious ideal, allowing for an adherent to utilize an emotional encounter as a means to develop a connection with his creator, it does not, in my humble opinion, work in other contexts. Indeed, even in the religious context, such channeling of thoughts and emotions is not simple and accomplished only by the truly devout (see, e.g., Maimonides, 1956: III:51; Horowitz, 1873: Gen. 46:29). To expect people to “reference” an other through a SR while in the midst of their everyday mundane lives is utterly impractical. To help us envision the idea, Wales analogizes the connection of ‘robot-creator(s)–to–robot’ to that of ‘baker-to-cookie’—i.e., we could “reference” the baker when we eat his cookie. It is certainly nice to contemplate such a notion, but again, utterly impractical. Furthermore, I think a better analogy of ‘robot-creator(s)–to–robot’, instead of ‘baker-to-cookie’, would be ‘parent(s)-to-child’. This analogy, I believe, makes clear just how terribly difficult it is to redirect or refer one’s thoughts to an other—for, can one really focus on the parent(s) of a child while interacting with the child alone—whether upon first thought or, as Wales suggests, upon second thought.^17^ Again, as a religious ideal, reflecting upon the creator in an encounter with an other may be a worthy challenge, but to import the technique to robot encounters will simply not work.^18^

An opposing attempt to resolve our dilemma is brought by Raffaele Rodogno (2016). That is, if Wales attempted to solve the dilemma by framing the HRR as very real, the solution offered by Rodogno is to cast it as utterly fictional:

… we could hypothesize that, when engaging affectively with robot pets, individuals adopt a cognitive mode akin to that which is normally adopted in our engagement with fiction. Being emotionally engaged by robot pets would be akin to being emotionally engaged by a good novel or movie. Just as my sadness for Anna Karenina involves my *imagining*, *accepting*, *mentally representing* or *entertaining the thought*, *without believing*, that certain unfortunate events have occurred to her, my joy at the robot pet involves my imagining, accepting, mentally representing or entertaining the thought, without believing, that it is happy to see me (Rodogno, 2016: 11).

This solution is untenable for a number of reasons. First of all, the relationships we build with fictional characters on the page or screen are both temporary and passive—our interaction with them is limited in time and confined in “space” to our own mind. Robot interactions, in contradistinction, are ongoing active relationships with entities deceivingly alive in the three dimensional space in which we live. As such, they are very different not only from fictional storybook characters but even from real dolls that are not animated to the point that we ascribe to them beliefs, desires and intentions (see, e.g., Turkle, 2011a: 39). Secondly, as noted above (sec. 2 The Dilemma), we take these relationships quite seriously, treating them as if they were not merely fictional—a fact that has dangerous consequences, as Gerdes notes: “the relational *as if* approach is challenged by the fact that, over time, our human-human relations may be obscured by human-robot interactions” (Gerdes, 2016: 276).

In psychological terms, the HRR engenders a state of cognitive dissonance (Festinger, 1957) wherein one knows he is interacting with a very real entity, a SR, while at the same time knowing very well that the interaction is not “real,” not authentic. Both Wales and Rodogno attempt to diffuse the dissonance, but from opposite ends. Wales attempts to achieve cognitive harmony by relating the relationship to something real, authentic. That is, since the physical interaction is real, he tries to make the metaphysical relationship real as well. It doesn’t work because the referred metaphysical relationship can’t be imagined. Attacking the problem from the other end, Rodogno attempts to achieve cognitive harmony by framing the relationship as completely fictional, inauthentic. That is, since the metaphysical relationship is fictional, he tries to make the physical relationship fictional as well. It doesn’t work because the physical relationship can’t be imagined away.

## Virtuous Servant Owner

And so we return to our question: How are we to relate *morally* to social robots?

Having reviewed the various attempts to construct a response, it is clear that the question, in both physical and metaphysical terms, is strained in the tension between the need to preserve virtue, on the one hand, and the need to preserve authenticity, on the other—what might be termed the Virtue-Authenticity Dialectic (VAD). The ideal response, then, must strive to allow us to maintain our virtuous character, such that we not act in dehumanizing ways toward SRs, but at the same time allow us to maintain our appreciation for authenticity, such that we not accustom ourselves to “as if” relationships *as if* they were real.

As for the “virtue” part of the response, Aristotle’s virtue ethics, as echoed in Kant’s appeal to indirect duties toward animals, soundly satisfies this need as evidenced by its broad support among thinkers in the field. As for the “authenticity” part of the response, thinkers in the field, as noted, run into trouble.

To address the “authenticity” issue, it is instructive to revisit Aristotle’s approach to automata as found in his *Politics*:

Now of instruments some are inanimate and others animate—the pilot’s rudder, for example, is an inanimate instrument, but his lookout an animate one; for the subordinate is a kind of instrument whatever the art … if each of the instruments were able to perform its function on command or by anticipation, as they assert those of Daedalus did, or the tripods of Hephaestus (which the poet says “of their own accord came to the gods’ gathering”), so that shuttles would weave themselves and picks play the lyre, master craftsmen would no longer have a need for subordinates, or masters for slaves (Aristotle, 2013: 1253b).

Aristotle here envisions that automata will replace slaves as instruments of their masters (similarly, *Nichomachean Ethics* 1161b). Now, while Aristotle may have been the first to articulate this instrumental approach, the history of automata, real or fictional, leaves little doubt that automata were forever imagined to be slaves (see, e.g., LaGrandeur, 2013). And with the advent of AI they continue to be so imagined. Hans Moravec claimed, ‘By design, machines are our obedient and able slaves’ (Moravec, 1988: 100); Nick Bostrom argued that “investors would find it most profitable to create workers who would be ‘voluntary slaves’” (Bostrom, 2014: 167); but no one popularized the notion more than Joanna Bryson (2010) who entitled her article on the issue, “Robots Should Be Slaves.” Her claim received no small amount of pushback given the cultural scars left on society by the brutal history of human slavery (Bryson, 2020b).

And that brings us to the heart of the matter, for while it is clear that the goal of automation is to relieve humans of their burdens,^19^ slavery is an institution that runs counter to modern values. Slavery is an institution that, despite Aristotle’s justifications (*Politics*, Book 1, Chs. 4–5), has been shown to undermine the very virtue ethics that Aristotle sought to foster. Powerful evidence of this can be seen in the testimony of Fredrick Douglass (1845) who wrote of his experience as a slave under a woman he refers to here as “my mistress”—i.e., “female master” slaveholder:

My mistress was, as I have said, a kind and tender-hearted woman; and in the simplicity of her soul she commenced, when I first went to live with her, to treat me as she supposed one human being ought to treat another. In entering upon the duties of a slaveholder, that [now] I sustained to her the relation of a mere chattel, and that for her to treat me as a human being was not only wrong, but dangerously so. Slavery proved as injurious to her as it did to me. When I went there, she was a pious, warm, and tender-hearted woman. There was no sorrow or suffering for which she had not a tear. She had bread for the hungry, clothes for the naked, and comfort for every mourner that came within her reach. Slavery soon proved its ability to divest her of these heavenly qualities. Under its influence, the tender heart became stone, and the lamblike disposition gave way to one of tiger-like fierceness (1845: 32).^20^

Accordingly, as described previously, many have expressed concern that modern robots designed to serve humans will be treated as slaves and engender a moral calamity for their owners.

But is this outcome not unavoidable? Kant believed it is. He wrote that while one must not hold a slave because, in so doing, one violates the freedom that is at the essence of the individual as a person, nevertheless, one could come to an agreement into which the servant enters of his own freewill and can exit of his own freewill. In such a case, Kant, in his *Metaphysics of Morals*, writes:

Servants are included in what belongs to the head of a household, and, as far as the form (the way of his being in possession) is concerned, *they are his by a right that is like a right to a thing*; … But as far as the matter is concerned, that is, what use he can make of these members of his household, *he can never behave as if he owned them* (6:284. *Emphasis added*).^21^

Kant here claims that you can maintain a relationship in which, on the one hand, you are in the position of a servant owner; yet, on the other hand, your behavior toward your servant never expresses this position. I believe that we can reconcile Kant’s claim with the seemingly damning evidence brought by Douglass to the contrary, as follows.

Douglass wrote: “In entering upon the duties of a slaveholder, she did not seem to perceive that [now] I sustained to her the relation of a mere chattel, and that for her to treat me as a human being was not only wrong, but dangerously so. Slavery proved as injurious to her as it did to me.” That is, only upon fully accepting the slaveholder role—in which one relates to the slave as chattel and in which treating a slave as a human being is “not only wrong, but dangerously so”—does slaveholding becomes injurious to the slaveholder. The injury to the slaveholder, then, is when the slaveholder assumes that one must treat the slave as non-human. That is, it was not the owning of a slave per se, but the social concepts of the time that dictated *how* one needed to treat a slave—i.e., by force of “tiger-like” subjugation to ensure obedience.

A machine programmed for obedience, however, would never occasion its owner to impose her will. Nevertheless, there remains a further moral concern in owning a slave, humanoid or human:

There is some harm to one’s own higher moral values and moral character if one establishes oneself as master… The problem of using and treating machines as slaves is that one perpetuates a value that sustains the inappropriate agent character, seeing the world and its denizens as one’s slaves. You simply should not treat the world as a place in which your will is absolute. You thereby only strengthen that absolutist, disregarding will (Miller, 2017: 5; similarly Coeckelbergh, 2021: 7).

This harkens back to Kant’s dog and the concern against habituating vicious character through vicious behavior. In employing machine-slaves, as stated at the outset: we will likely not treat people as slaves, but we will certainly be in danger of treating people as objects rather than subjects. Accordingly, Kant is not concerned for the virtue (or loss thereof) of one who maintains a servant, as long as she behaves toward her servant as a human being and not as “a thing.” Sven Nyholm writes that “Kant himself thought that having a human servant does not need to offend against his formula of humanity [i.e., that one must treat others as ends and not merely as means]—so long as the servants are treated well and with dignity” (2020:192).

This idea finds precedence in the legal writings of the Medieval Jewish philosopher Moses Maimonides. He not only preceded Kant in demanding that servants be treated with dignity, he also elaborated such treatment with details that are instructive in both pragmatic and moral dimensions. Here is his original text (*Laws of Slaves* 8:9), interleaved with some clarifications of mine:^22^

*It is permissible to work a heathen slave relentlessly.* [Biblical law often promulgates rules in concert with ancient custom while nevertheless seeking to provide a moral improvement on the accepted state of affairs (see, e.g., Korn, 2002; Rabinovitch, 2003; Lamm, 2007; on slavery see, e.g., Shmalo, 2012). As such, the strict letter of law allows for slavery but with various moral restraints.^23^ The law, however, is seen as a starting point, a floor and not a ceiling, to use the words of Rabbi J. D. Soloveitchik. Accordingly, Maimonides starts with the legal “floor” only to show that we should—and must—rise far above it. It is interesting to note that Kant (*Metaphysics* 6:284) used the same format, starting with the letter of the law allowing for ownership only to then argue for virtue].

*Though this is the law, the quality of virtue and the ways of wisdom demand of a human being to be compassionate and pursue justice, and not make heavy his yoke on his slave nor distress him.* [Maimonides, here, raises us off the floor of the law, outlining his thesis that calls for virtue and justice. He will now elaborate on these two categories, bringing proof texts to support his claims].

*He should give him to eat and drink of every food and drink. The sages of old had the practice of sharing with the slave every dish they ate. And they would provide food for their animals and slaves before partaking of their own meals. As it is said, “As the eyes of slaves follow their master’s hand, as the eyes of a slave-girl follow the hand of her mistress, [so our eyes are toward the Lord our God, awaiting His favor]*.*”* [Here Maimonides provides concrete actions toward maintaining virtuous interactions, grounded in a verse equating master and slave in their shared neediness].

*Nor should a master disgrace his servant, neither physically nor verbally; the biblical law gave them to servitude, not to disgrace. And one should not treat him with constant screaming and anger, but rather speak with him calmly and listen to his complaints.*^24^ [Clearly the servant is not to be treated merely as a means but as an end. (I wonder if even Kant would have made such a list of directives to regulate the owner).] *This is explicitly stated with regard to the positive paths of Job for which he was praised: “Have I ever shunned justice for my servants, man or maid, when they quarreled with me… Did not He who made me in my mother’s belly make him? Did not One form us both in the womb?” (Job 31:13,15)*. [The claim here is for just relations, supported by the verse that notes the physiological identity of master and slave].

*Cruelty and effrontery are not frequent except with the heathen who worship idols. The progeny of our father Abraham, however, the people of Israel upon whom God bestowed the goodness of the law (Bible), commanding them to observe “righteous statutes and judgments” (Deut. 4:8), are compassionate to all.* [Maimonides defuses any claims that come to justify slavery merely because such treatment is “accepted practice” among the nations of the world. This is not some parochial diatribe against non-Jews,^25^ but rather part and parcel of his argument for just relations with one’s servant, here made irrespective of the inherent value of the servant. That is, justice is incumbent upon the master for the sake of his own virtue and character].

*Accordingly, regarding the divine attributes, which He has commanded us to imitate, the psalmist says: “His tender mercies are over all His works” (Psalms 145:9)*. [Here, as part of his thesis that one must move beyond the strict letter of the law in the treatment of one’s servant, Maimonides reminds us of the ethical imperative to strive to imitate the divine virtues, chief among them being that of mercy/compassion. This claim, like the previous one, is incumbent upon the master irrespective of the inherent value of the slave. Worthy of note is that the support verse does not say that God’s “mercies are upon all His creatures” but “upon on all His works.” Could this not be understood to allow for application to humanoids?]

*Whoever is merciful will receive mercy, as it is written: “He will be merciful and compassionate to you and multiply you” (Deut. 13:18)*. [Maimonides concludes his call for virtue with a religious principle known as “measure for measure,” which states that in the measure, or manner, that you act towards others, so too, in the same measure, will God act towards you. Accordingly, even if one does not appreciate the value of a virtuous character, one will certainly appreciate the selfish need of God’s mercy. In addition, this call to mercy, to virtue, is made independent of the worth of the servant. It pleads for virtue saying: though you may not recognize the worth of your servant, nor even the worth of your own character, at least recognize your need for mercy and be merciful.]

This text stands as a powerful call to virtue in general, and to virtuous behavior with one’s servant in particular. Maimonides here speaks to any and all, regardless of what “stage on life’s way” one might be. Indeed, his arguments for virtuous behavior can be seen as addressing the individual in each of the three Kierkegaardian stages of existence, stages in which one is driven by the corresponding motivations: aesthetic, ethical and religious.^26^ Starting with the ethical, being that it is the universal—applying to all and in which all struggle (Kierkegaard, 1985: 83), Maimonides enjoins virtue based on the human dignity inherent in the servant as a human being. Moving to the higher motivation of the religious, Maimonides calls for the master to exhibit virtue both because he is a God fearing individual who, like Abraham,^27^ accepts the divine ethical norms of the Bible and furthermore, because he is to emulate the attributes of the creator, mercy being primary among them.^28^ Maimonides concludes with an appeal to self-interest (i.e., the Kierkegaardian aesthetic), arguing, in essence, that even if one is not moved by these higher motivations, one should act mercifully that he too will be treated mercifully.

Not satisfied in leaving his readers with “mere” motivations, Maimonides takes pains to prescribe practical action. He instructs the master to feed his slave with “every dish” that he himself eats, thus raising the slave to the dignity of the master. He directs the master to feed his slave before he himself sits to eat, thus instilling compassion toward he who is not in charge of his own food. He warns the master to “speak calmly and listen to the slave’s complaints,” thus changing the very relationship from one of master-slave to one more akin to employer-employee (and a quite considerate employer at that). Maimonides thus transforms ethical ideal into ethical practice which, ultimately, shapes ethical character (Aristotle, [350 BCE] 2004: 23; Ha-Levi, [1523] 1978: Precept 16; Vallor, 2018: 3.3; Cappuccio et al., 2019; Coeckelbergh 2020b).

Of course no ownership, no matter how virtuous, can be justified today. Slavery is an institution that is anathema in modern moral thought and given circumscribed sanction in the bible, due only to ancient cultural mores. Jewish thought has ever sought to ameliorate the master-slave relationship (see, e.g., Shmalo, 2012) to the point that Maimonides demands not simply that one treat his servant as an end, but that one treat him as nothing less than a contemporary! He does so, as mentioned, by providing clear practical behaviors underpinned by clear philosophical reasoning, (albeit) based on biblical verse. Significantly, his arguments are not found not in his philosophical writings but in his legal writings, thus giving them normative import and evincing, essentially, a law to go beyond the law.

And this brings us back to SRs. My point here is not to argue for even this most virtuous form of human slavery, but to apply the Maimonidean paradigm—what I call the “Virtuous Servant Owner” (VSO)—to Human Robot Relationships. For, though the virtuous practices demanded by Maimonides address, in part, the biological needs of a human servant (e.g., *feed the servant every dish the master is fed*), the practices, in general, express the need for dignity, compassion and consideration—practices that every virtuous individual must pursue, whether his interlocuter is human or, as is my thesis, humanoid. Accordingly, while *feeding the servant first* is not relevant, saying “please” and “thank you” is relevant, part and parcel of the requirement to *speak calmly*. Similarly, while *feeding the servant every dish the master is fed* is inapplicable, not raising one’s voice in anger nor one’s hand in violence is most applicable, falling under the rubric of *not disgracing the servant verbally or physically*.

It is my contention that this master-slave relationship delineated by Maimonides provides an eminently reasonable paradigm for interacting with the social robot, one that can provide a resolution to the VAD (as well as the ADP). Starting with the “virtue” part of the “Virtue Authenticity Dialectic”, the VSO model demands that we abide by the highest ideals of a virtuous relationship, thus distancing us from the dehumanization trap. This, of course, is the approach taken by Cappuccio et al., and really the whole “appearances” camp, which leads to the problems associated with anthropomorphizing. However, whereas Cappuccio et al., shun the slave-like relationship as “disturbing,” VSO embraces it in virtue. VSO defines the SR as our slave, our property, our instrument, all the while commanding us to behave virtuously with it, treating it as an end. Relating to the SR not merely as an instrument, but as an end, allows us to maintain our own virtuous character. Keeping the SR on the level of instrument, allows us to avoid bringing it in to our moral circle and thus avoid *most* of the Pandora’s box of misplaced moral status issues.

I say “most” because we are still left with the “authenticity” part of the “Virtue Authenticity Dialectic.” That is, if we are interacting with the SR as an end, treating it in the most virtuous of ways, we will, in the words of Turkle, “imagine that the robot is an ‘other’”—i.e., a being to engage with emotionally. How, then, can we retain our appreciation for authentic, reciprocal, relationships—relationships in which both parties understand, in the deepest sense, what they themselves are thinking, saying, and doing?^29^ How can we remain cognizant of the value of mind-ful humans over mind-less humanoids?

I suggest that it is precisely by framing the relationship in terms of master-slave that we maintain our distance and are ever brought back to the reality that we are interacting with a machine and not the noblest of creations—a conscious human being. The VSO paradigm holds that, while we maintain a virtuous relationship with the SR, we nevertheless bind that relationship in the rubric of master-slave. In so doing, we are forced to abandon the thought that we are having an authentic relationship for the simple reason that such would imply we are, in fact, slaveholders! This would then implicate us as being in violation of the fundamental principles we hold dear: freedom and equality for all humanity. It is, then, the very designation—“slave”—that awakens in us the realization that the relationship with the SR is not authentic, that “insides count” and that authenticity is precious, to be found only in conscious beings.

And is this not what the name robot was supposed to denote from its very beginning? Karl Capek coined the name robot from the Czech word robota meaning “forced labor.” But the name robot has since lost its original intent and so a more telling appellation is of the essence. “Slave,” though repugnant to modern ears, is really the term that drives home the idea of the robot, for it is precisely this repugnance that allows us to use the SR as the tool it was made for and not as the friend it appears to be.^30^ Nevertheless, due to the negatively charged nature of the term (see, e.g., Miller, 2017: 298, Gunkel, 2018: 131), I suggest we use the “less polarizing” term, to quote Gunkel (ibid.: 130), of “servant.” And while thinkers such as Coeckelbergh (2015: 224) question if there is a difference in the terms, I believe there is a world of difference—one that turns on Kant’s prescription for human relations. Slave implies chattel, treated as a mere means. Servant implies worker, with the potential to be treated as an end (see, e.g., Bryson, 2010). Slave, according to Steve Petersen (2007: 45), implies working against one’s will; servant implies *wanting* to work. Certainly a mindless humanoid cannot be considered as working against its will, for it has no “will,” and though it similarly has no “wants,” by being programmed to serve it could be considered, anthropomorphically, as *wanting* to serve.^31^

## Getting the Metaphor Right

That said, whether slave or servant, the metaphor has given rise to numerous objections. Objections that, as Joanna Bryson has contended in her now infamous piece “Robots Should be Slaves,” eventuate from failure to “get the metaphor right.” By this she refers to the fact that metaphors are imprecise. We use metaphors as tools, conceptual tools, that allows us to think about things we don’t know by comparing them to things we do know. But metaphors, by definition, are limited—“there is an apparent claim of identity, but … only with respect to certain characteristics” (Ortony, 1975: 52; see also, Jones and Millar, 2017: 604). Accordingly, the slave metaphor is to be used to address the question of the moral interaction with mindless humanoids not as if it entailed identity but only as a rough conceptual paradigm.

And this is where thinkers, as described by David Gunkel, run in to trouble; for, in the effort to demonstrate that robots should not be slaves, that the slave metaphor “may be the wrong metaphor” (2018: 131), the metaphor is assumed to entail identity—i.e., that what is true for human slaves is true for robots. To take but one example, it is explained that slaves have criminal responsibility in Jewish, Roman and United States law, yet applying this to robots is problematic since punishment works only if something matters to the punished (ibid: 123-5). The metaphor is thus stretched to imply its failure. But “getting the metaphor right” means applying it judiciously.

Bryson (2020b) herself writes: “The mistake I made with that title [“Robots Should be Slaves”] was this belief that everyone was sensitive to the truth that you can’t own people. The word slave here is about something else.” That is, the metaphor only goes so far, robots are to be slaves in the sense that their function is to serve human needs and in the sense that they have no responsibility for their actions and in the sense that we have no direct moral responsibilities toward them (similarly, Grau, 2011: 458).

Veruggio and Abney note that, indeed, it is impossible to apply all of the moral implications latent in the term “slave” to mindless humanoids, for “in reality, our robots are not (for now, anyway) our ‘slaves’ *in any robust sense*, as they have no will of their own” (Veruggio and Abney, 2012: 352, *emphasis added*). Again, any use of the term “slave” can only be applied in a very limited sense—as found, for example, in computing terminology wherein slaves and masters are simply logic agents, the former accepting and executing commands at the request of the latter.

Veruggio and Abney explain that we view our relationship with robots incorrectly, incoherently, because we are “driven by our collective guilt over the history of slavery” (ibid). Now, while numerous authors have used this guilt driven approach to argue against the slave metaphor (see, e.g., Lavender, 2011; Dihal, 2020) no one has argued the point more obdurately than Gregory Jerome Hampton (2015). Hampton begins by noting that the motivations for robots are the same as for slavery—i.e., cheap labor requiring the “human” touch, one that combines intelligence and dexterity. Though this is true enough, he extrapolates from here to argue that the deployment of robot slaves is identical to the deployment of human slaves. The claim is fallacious because, as Veruggio and Abney noted, robots have not a will of their own.^32^ The deployment of mindless humanoids, then, is more like the deployment autonomous cars—the likes of which no one imputes with slavery.

Hampton goes on to express the fear, without providing support, that the deployment of robot slaves will prompt racism. Now, while there is a concern that mistreating robots that impersonate a specific race (or gender) will “confirm and proliferate” such behavior in society at large (Coeckelbergh, 2021: 7), it is hard to see why racism (or misogyny) would emerge otherwise—i.e., without mistreatment or without impersonation. That said, it could be argued that speciesism against robots could emerge, for people do unfortunately harbor ill will toward the other (see, e.g., Gunkel, 2012: 207; Kim and Kim, 2012; Scheutz, 2014a: 249, Musiał, 2017: 1093). But even if speciesism were to result from deploying robot slaves, there is no reason to believe that this speciesism would prompt racism. Peter Singer (2009), who argues that humans exhibit speciesism against animals, does not argue that it has prompted or contributed to racism. He does say that all such prejudices are “aspects of the same phenomenon”—i.e., unjustifiably maintaining oneself as superior over an other (Yancy and Singer, 2015). So one could raise the concern that relating to mindless humanoids as slaves will inculcate a vicious character that could harden us, to echo Kant once again, in our interactions with human beings in general, but not toward one race in particular. But this concern over inculcating a vicious character is one that has already been raised and addressed directly by the VSO paradigm which demands virtuous behavior toward humanoids (as explained in the VSO section above).

Another claim against deploying humanoid robots as slaves is made by Kevin LaGrandeur (2011: 237) who applies Aristotle’s warning to beware of powerful slaves who will revolt. That is, once slaves become more powerful than their masters—be they human or humanoid—they will revolt. This may be an issue for “strong AI,” as LaGrandeur states, but a mindless humanoid, while more powerful than humans in many respects, does not have an autonomous will to revolt, indeed, does not have an autonomous will period. Accordingly, this concern is of no consequence with respect to mindless humanoids.

That said, LaGrandeur argues that the mere interdependency of slave-systems with their human operators gives rise to what could be considered a “slave revolt” in the sense that the systems are delegated so much control that humans no longer control or even understand what the slave-systems are doing. We are reminded here of Hegel’s master-slave dialectic in which masters, by dependence on their slaves, lose touch with reality (Hegel, [1807] 2019). Mark Coeckelbergh, in his “The Tragedy of the Master: Automation, Vulnerability, and Distance” (2015), applies this dialectic to automation in general, and to AI and robots in particular, explaining that robots as slaves will bring upon us the tragedy of which Hegel warned: dependency on automation and alienation from nature. While this may indeed be true, it is neither a reason to stop the advance of automation nor to dissuade use of the master-slave paradigm. For, though the robot as slave, as with all automation, may bring dependency and alienation, it will also provide the boon of freedom from all the burdens inherent in taming nature to human needs. And employing the robot as slave will no more entail these negative “Hegelian” consequences than relating to the robot as companion—in any case, the very automation will engender dependency and alienation. That is the price of freedom from our burdens.

Additionally, Coeckelbergh (2015) argues against using the slave metaphor for we thus limit “the range of human–technology relations” when there are “different roles for, say, robots.” While clearly there are many roles robots can play, in speaking about SRs, they all assume human-like roles—whether as care-takers of the elderly, cleaning maids, teachers or hotel concierge—and they all accommodate the servant metaphor without inappropriately reducing the range of relations. The only role that the slave metaphor limits is “companion,” and this role, I believe, is one that should be proscribed. For, engaging socially with robo-companions may lead to the social catastrophe of shunning human companions, as Turkle notes, because they are “sometimes messy, often frustrating, and always complex” (2011a: 7, 295; see also, e.g., Richardson, 2015: 12; Gerdes, 2016: 277; Bertolini, 2018: 653).

Now, while many of the above arguments against using the slave metaphor are based on the “dehumanizing” nature of the term, Birhane and van Dijk (2020) argue that the metaphor should be eschewed because it “humanizes” the machine. That is, the term “slave,” while clearly dehumanizing when applied to mind-ful humans, is paradoxically humanizing when applied to mindless humanoids. By calling a robot a “slave,” they claim, we employ a term reserved for humans and thus implicitly make it human; and as a result, we then find ourselves in the immoral position of a slaveowner. To their claims I have two responses. First, the term does not serve to humanize the humanoid any more than our own natural anthropomorphizing of it does—i.e., in any case, as noted above, we “humanize” it. Second, and more to the point, the fact that we will find ourselves in the immoral position of slaveholder is a welcome implication, as explained previously, that forces us to abandon the illusion that we are interacting with a human being, loathe as we are to be found in violation of the freedoms of a conscious being.

One might counter that many (or most) people will not be so loathe. Yet this is precisely what VSO comes to address. VSO is to be seen as a kind of “user instruction manual” requiring the user/owner to relate to their humanoid servant in a virtuous manner. And while a user manual is no guarantee against user abuses, given that VSO requires the master to “*listen to the complaints*” of his servant, VSO concomitantly requires that the humanoid itself be programmed to provide moral feedback/pushback, reminding the master of his duties (similarly, Darling, 2016; Cappuccio et al., 2020). One can imagine an abusive owner screaming epithets while their robo-servant calmly objects with rational feedback. Will this tame the beast? The answer is irrelevant because such an interchange already removes Birhane and van Dijk’s objection that the human will become a slave owner. For, a slave, in the face of such abuse, would cower in submission not persist in moral exhortations and refusal to comply. Accordingly, without an obsequious entity to comply, there is no position for an immoral slaveowner to occupy.

This could, however, lead to the master becoming so frustrated that he “kill” his robo-servant. But there can be no “killing” of a mindless machine, only a powering down. Interestingly, it was precisely due to this moral fallacy that Bryson originally applied the slave metaphor. Shocked that people expressed repugnance at the idea of turning off a mindless humanoid, she went on a campaign to decry the notion that a mindless humanoid had moral patiency (Bryson, 2016). When her efforts failed, she decided to employ the slave metaphor to emphasize that we *can* turn humanoids off. She did not mean to imply that we can kill human slaves but only that we must realize that the humanoid robot is built to serve, that they are, in her words: “tools to extend our abilities and increase our efficiency in a way analogous to the way that a large proportion of professional society, historically, used to extend their own abilities with servants” (2010). The servant metaphor, then, was meant to be applied in the sense that mindless humanoids are like servants functionally, i.e., in the operations they perform. It was not meant to humanize nor to imply an identity to human slaves, and though there is admittedly ambiguity here, she meant just the opposite—i.e., the mindless humanoid has not rights nor feelings nor anything human-like that would engender moral patiency. That, she explains, is “getting the metaphor right.”

## Conclusion

In this essay I have taken up the most unpopular position of defending the indefensible: slavery. Of course, I am in no way, shape, or form, advocating human slavery but rather appropriating the paradigm, the metaphor, if you will, in its most virtuous form to guide human interactions with mindless humanoids. I have taken this position, despite the opposition voiced in much of the philosophic community, because I believe that human authenticity, human worth, and human-human relationships are at stake. If we do not appreciate that we are more than “meat-machines” and that our relationships with each other are more than instrumental, we will fail ourselves as human beings and usher in a world of untold moral calamity. It is a category mistake to equate man and machine. The VSO paradigm counters this mistake by maintaining a clear distinction between man and machine, all the while asking man to cultivate virtue in his interaction with machine.

Does this resolve the dilemma inherent in the Virtue-Authenticity Dialectic? As mentioned before, dilemmas are so designated because they have no perfect resolution. I admit that it is problematic to call an entity that appears human-like a “slave,” or even, a “servant.” I admit that engaging with human-like SRs makes it difficult to disassociate them from real humans. Nevertheless, given the options, I suggest that being a Virtuous Servant Owner allows us to maintain our own virtuous disposition on the one hand, while preserving our appreciation for human authenticity and authentic relationships, on the other.

Accordingly, whereas Cappuccio et al. sought a way to remove the “alienating representations of slavery,” I suggest that it is specifically this alienation that is redeeming. It can allow us to define a new ontological category, not human, not animal, but slave/servant—i.e., animated autonomous tool. And we need not fear the reinstitution of human slavery, for with the introduction of robots as animated autonomous tools, we will eliminate any advantage of human slaves—exactly as Aristotle envisioned.^33^

## Data Availability

The original contributions presented in the study are included in the Article/Supplementary material, further inquiries can be directed to the corresponding author.

## Footnotes

1 For the sake of completeness, it should be made clear that Aristotle did envision *intelligent* artificial servants, nevertheless, he could not imagine interacting with them other than as natural slaves, since slaves were a natural part of his politics. His desire for automatons was motivated not by ethical qualms but by expediency (*Politics* 1253b). For more on this see LaGrandeur (2013: 9–11, 106–108).

2 For the sake of completeness, today’s AI is known as Narrow or Weak AI, which uses algorithms to analyze data, mathematically, and make decisions accordingly. This is as opposed to General or Strong AI (sometimes referred to as GAI or AGI), which seeks to make machines intentional with consciousness. How will this be done is of great debate. There are “computationalists” (e.g., Ray Kurzweil, Hans Moravec) who believe that when every brain function is implemented at the level of human brain processing power, consciousness will “emerge.” Others (e.g., Pentti Haikonen) explain that it is not just the computational power that is needed but the way the computations are done (e.g., via associative neural networks, etc.). Still others (e.g., Roger Penrose, Colin Hales) believe that computation in itself, in any manner, is not enough but rather the physics of the brain must be replicated for consciousness to emerge.

3 While there is much to be said in regard to our moral attitude toward sentient robots, such a discussion remains outside the scope of this article.

4 I make the proviso of “humanlike” to exclude autonomous mobile computers like autonomous vehicles or assembly-line machinery for which I have yet to read of individuals becoming emotionally engaged.

5 For a concise discussion of the is-ought debate see Gunkel (2018: 3–4). See also Coeckelbergh (2013: 63), Schwitzgebel and Garza (2015: 99).

6 The term applies whether the SR is driven by conventional programming (i.e., rule based hard-coded algorithms) or machine learning (see, e.g., Domingos, 2018; Boucher, 2019).

7 The debate on whether it is possible to give machines emotions and feelings is outside the scope of this paper. Suffice it to say that truly sentient machines are not, as mentioned above, in the offing.

8 Kant famously held that the line dividing those deserving of moral status versus those undeserving of such was “judgement” (or reason), a position which became anathema following Bentham’s revision of the dividing line to “sentience,” or more precisely, the ability to suffer (Bentham, [1789] 2019). So, while Kant’s example of dog may grate on today’s sensibilities, it provides a fitting paradigm to address the mindless humanoid which has neither judgement nor sentience.

9 Worthy of note is that Kant (1724–1804), here, was preceded by Nachmanides (1194–1270) who explains that the biblical command to send the mother bird away before taking her eggs was promulgated in order “that we should not have a cruel heart and lack compassion … and is to prevent us from acting cruelly” (Nachmanides, 1976: Deut. 22.6). Thus, while some argue that Kant’s words point only to a concern for causal action and not character disposition (see fn. 10 herein), Nachmanides explicitly voices concern for both aspects, reiterating, “the reason for the prohibition is to teach us the trait of compassion and that we should not be cruel … ” (ibid.).

10 Worthy of note is the disagreement over whether Kant is concerned only with the externally causal effect—e.g., kicking a dog will bring one to kick a human (see, e.g., Coeckelbergh, 2020b; Coeckelbergh, 2020c; Sparrow 2020)—or does Kant’s demand for virtuous behavior because it reflects on the character of the individual (see, e.g., Gerdes, 2016; Denis, 2000).

11 Many make this argument, e.g., Bryson (2010: 65), John McCarthy and Marvin Minsky in Metzler (2007: 15), Miller (2010), Grodzinsky et al. (2014), Schwitzgebel and Garza (2015: 113), Richards and Smart (2016: 21) and Leong and Selinger (2019). The position is even offered as a regulatory principle (Boden et al., 2010: #4), though Wales (Gunkel and Wales, 2021: 11) argues it will simply not be followed.

12 Many make this argument, e.g., Breazeal (2002), Duffy (2003), Walker (2006), Darling (2017), and Burdett (2020).

13 Cappuccio et al. (2019: 10) note the two camps explicitly; so too, Torrance (2013: 10). Gunkel (2017, 2018) adds two additional camps in order to account for sentient machines (which, as mentioned, are beyond the scope herein). It should be noted that Gunkel defines yet another camp for himself.

14 This approach is found in numerous authors, as, for example, the following list shows. Coeckelbergh (2010b): a robot is “quasi-alterity” to be treated as it appears to us. Burdett (2020): a robot is “quasi-person” which demands “Thou” relations. Don Ihde (1990: 100): a robot is “quasi-other” but remains lower than human or animal; see also Bergen and Verbeek (2020). Peter Asaro (2006): a robot is “quasi-moral agent” giving it some level of responsibility. Philip Brey (2014) argues that the term “quasi-moral agent” denotes involvement in moral acts but without true moral responsibility. Gunkel (2018: Ch. 6) argues for Levinasian alterity relations—i.e., a robot is a full other, not simply a quasi-other.

15 This is a well-known religious technique wherein one is to channel one’s emotions toward God in an effort to connect to the source of all emotion and life itself (see, e.g., Horowitz, 1873: Gen. 46:29).

16 Burdett (2020: 355), basing himself on Pattison, makes a similar point. All of these thinkers have been preceded, in a sense, by Buber (1970: 175) who, upon confronting a Doric column in a Syracuse church, writes that he related to the “spiritual form there that had passed through the mind and hand of man and become incarnate.” A distinction worthy of note is as follows. Buber is seeking to establish the I-Thou relationship with the inanimate by “referring” to the humanity behind it—he is trying to generate a close, “Thou”, relationship; while Wales is trying to “refer” the already close “Thou” relationship to its underlying humanity to avoid seeing the robot as more than it is and falling into the misplaced-empathy trap.

17 Wales attempts to make the creators of the robot more resident in the robot by explaining that it is not the engineers who built the robot that are represented in the robot, but the very people whose behaviors made up the data that was used to train the neural network that grounds the robot’s behaviors. However, the same could be said of the child whose behaviors are made by the DNA and parental education that make up the neural network that grounds the child’s behavior. In any case, the notion of referencing is not practical.

18 I make this claim as a religious man who appreciates the religious ideal. I am not alone in this claim, for when I made it directly to Wales at the RP2020 conference (as he notes in his fn. 22), many other voices joined me in dissent and none his in defense.

19 There is a vast literature on how automation, and specifically AI, will replace human labor, see, e.g., LaGrandeur (2013: 161), Marr (2017), Harari (2019: Ch. 2), and Coeckelbergh (2020a: 136).

20 Similarly, this slave girl testimony: “I can testify, from my own experience and observation, that slavery is a curse to the whites as well as to the blacks. It makes the white fathers cruel and sensual; the sons violent and licentious; it contaminates the daughters, and makes the wives wretched” (Jacobs, 2020); as well as that of French philosopher Alexis de Tocqueville, “Servitude, which debases the slave, impoverishes the master” (de Tocqueville [1835] 2013).

21 An important aside: Kant’s contract binds the servant but nevertheless allows him to quit. The servant is then like a slave in the sense that he is the property of, and at the command of, the owner, all the while retaining some human dignity in his ability to exercise his will to both enter and exit the contract freely. In reality, however, it would seem that someone in a position to accept such a contract would be in such dire straits that he will likely never have the means to exit the contract. As such, he is only a “free” servant in name but a slave in practice. Furthermore, it is not clear how the owner can unilaterally, according to Kant, “fetch servants back” (ibid.), if the servants are allowed to terminate the contract at will. The only way this makes sense is by saying that the servant failed to give notice when he left. But why would he not give notice and leave legally if he could do so at will? Maybe the giving notice of leave is actually very limited. It seems that Kant’s ownership is closer to slavery than would at first appear.

22 A detailed analysis of this text is being prepared for publication by the author.

23 For example, killing a slave entails capital punishment (Ex. 21:20, Rashi ad loc.), a slave is set free if injured (Ex. 21:26-27, Kid. 24a), a slave rests on the Sabbath (Ex. 20:9); a runaway slave is not to be returned (Deut. 23:16). On the differences between ancient slavery versus that of the Torah, see Beasley (2019).

24 Interestingly, in terms of a model for SRs, this would demand that the SR give negative feedback, and as Kate Darling suggests, “respond to mistreatment in a lifelike way” (Darling, 2016: 228; similarly, Cappuccio et al., 2020).

25 Worthy of note is the great esteem in which Maimonides holds non-Jewish thinkers, frequently quoting, Aristotle and Al Farabi.

26 Worthy of note is that Maimonides (1956, 3:33) appears to refer to these categories in articulating the “ultimate causes of the Law”: 1) the rejection and reduction of the fulfillment of desires—i.e., aesthetic, 2) the promotion of virtuous interaction between men—i.e., ethical, 3) the sanctification of its followers—i.e., religious.

27 Like Kierkegaard, Maimonides references Abraham as the father of faith; yet unlike Kierkegaard, Maimonides, indeed Judaism in general, does not accept the notion of a religious leap of faith as requiring a teleological suspension of the ethical (see Navon, 2014).

28 The two demands could be seen to reflect the two levels of the “religious” articulated by Kierkegaard (see Broudy, 1941: 306).

29 On the importance of authentic reciprocal relationships, see, e.g., Turkle (2011a: 6, 2011b: 64), Richardson (2016: 51), Prescott (2017: 143), Bertolini and Arian (2020), and Nyholm (2020: 111–2). Similarly, Veruggio and Abney (2012: 355).

30 And marking the SR as non-human, or even making it look completely non-human, is untenable because of the great advantages to having them as humanlike as possible (see, e.g., Scheutz 2014b: 209; Ghiglino and Wykowska 2020: 55).

31 It should be noted that Petersen argues for the moral legitimacy of engineering mind-ful humanoid servants whereas I am merely discussing mind-less humanoids. Elsewhere (Petersen, 2017) he notes that mindless robots certainly have no moral patiency.

32 One could argue in his defense that he is, in fact, referring to mindful robots, however he writes explicitly that he refers to “anything resembling an independent consciousness” (2015: x), which readily includes mindless humanoids, as noted in my Introduction.

33 Note that even Mark Coeckelbergh (2015: 227) admits this point.
